# Association between milk and yogurt intake and mortality: a community-based cohort study (Yamagata study)

**DOI:** 10.1186/s40795-021-00435-1

**Published:** 2021-07-14

**Authors:** Akiko Nakanishi, Erika Homma, Tsukasa Osaki, Ri Sho, Masayoshi Souri, Hidenori Sato, Masafumi Watanabe, Kenichi Ishizawa, Yoshiyuki Ueno, Takamasa Kayama, Tsuneo Konta

**Affiliations:** 1 Department of Public Health and Hygiene, Yamagata University Graduate School of Medical Science, 2-2-2 Iida-Nishi, Yamagata, 990-9585 Japan; 2 Global Center of Excellence Program Study Group, Yamagata University Faculty of Medicine, Yamagata, Japan

**Keywords:** Dairy products, Milk, Mortality, Yogurt

## Abstract

**Background:**

Dairy products are known as health-promoting foods. This study prospectively examined the association between milk and yogurt intake and mortality in a community-based population.

**Methods:**

The study population comprised of 14,264 subjects aged 40–74 years who participated in an annual health checkup. The frequency of yogurt and milk intake was categorized as none (< 1/month), low (< 1/week), moderate (1–6/week), and high (> 1/day) intake. The association between yogurt and milk intake and total, cardiovascular, and cancer-related mortalities was determined using the Cox proportional hazards model.

**Results:**

During the follow-up period, there were 265 total deaths, 40 cardiovascular deaths and 90 cancer-related deaths. Kaplan–Meier analysis showed that the total mortality in high/moderate/low yogurt intake and moderate/low milk intake groups was lower than that in none group (log-rank, *P* < 0.01). In the multivariate Cox proportional hazard analysis adjusted for possible confounders, the hazard ratio (HR) for total mortality significantly decreased in high/moderate yogurt intake group (HR: 0.62, 95% confidence interval [CI]: 0.42–0.91 for high intake, HR: 0.70, 95%CI: 0.49–0.99 for moderate intake) and moderate milk intake group (HR: 0.67, 95% CI: 0.46–0.97) compared with the none yogurt and milk intake groups. A similar association was observed for cancer-related mortality, but not for cardiovascular mortality.

**Conclusions:**

Our study showed that yogurt and milk intake was independently associated with a decrease in total and cancer-related mortalities in the Japanese population.

**Supplementary Information:**

The online version contains supplementary material available at 10.1186/s40795-021-00435-1.

## Introduction

Dairy products such as milk and yogurt are known as health-promoting foods. However, the association between intake of dairy products and health outcomes has not been established. Several meta-analyses have shown that total dairy or milk intake was not significantly associated with total mortality, incidence of cardiovascular disease [1, 2], and cancer-related death [3]. However, some fermented dairy products, such as cheese and yogurt, showed a slightly reduced risk for total mortality and cardiovascular events [1, 4, 5]. These observations indicate that the association between dairy products and prognosis may vary depending on the type of dairy product consumed.

The consumption of dairy products is lower in Japan than in other countries with similar economic levels [6]. In some studies, the intake of dairy products was associated with a better prognosis. In a previous study, increased milk consumption was associated with a reduced risk for total, cardiovascular, and cancer-related mortalities in men [7]. In another study, increased yogurt intake was associated with reduced deaths due to rectal cancer in men [8]. Furthermore, increased weekly intake of dairy products has been reported to be associated with decreased cardiac mortality rates in women [9]. However, some studies have shown contradictory results. In a previous study, high frequency of milk and butter intake was associated with higher mortality due to blood cancer [10]. In another study, high intake of dairy products was associated with an increased incidence of prostate cancer [11]. Therefore, the association between dairy products and prognosis in the Japanese population may differ depending on the type of outcome.

The possible reasons for these inconsistent results might be the differences in the analytical methods such as classification of dairy products (total amount, fermented/non-fermented type, and individual products), assessment of the amount of intake (frequency or volume), background factors of study populations (age, sex, and comorbidities), and type of outcome (total or specific cause of death). Therefore, to clarify the association between dairy products and prognosis, it is necessary to examine the association between the type of dairy products and the cause of deaths in detail.

The Yamagata study aimed to investigate the effects of major genetic and environmental factors on the development of common diseases and death in a community-based population. This study provides detailed information on dairy consumption including milk and yogurt; background factors including age, sex, and comorbidities; and mortality during the 9-year follow-up period and causes of death.

In this analysis, we examined whether the intake of milk and yogurt, two major dairy products, is associated with mortality and cause of death in a Japanese population considering the environmental factors using the database of the Yamagata study.

## Methods

### Study subjects

The study population comprised 19,231 subjects aged 40–74 years in seven cities in Yamagata Prefecture (Yamagata, Sakata, Kaminoyama, Tendo, Sagae, Higashine, and Yonezawa) who consented to the Yamagata Study during the baseline survey between 2009 and 2015. Details of the Yamagata study have been described in a previous study [12]. The Yamagata Study was a community-based prospective cohort study, supported by the regional characteristics of the twenty-first Century program and Global Center of Excellence programs in Japan. The target population of this study was the local population covered by national health insurance. All subjects in this study provided written informed consent to participate. Of the 19,231 subjects who consented to the study, 14,264 subjects were included in the analysis after excluding those who withdrew their consent and those with missing responses to the questions on yogurt/milk intake and baseline characteristics (*n* = 3387) and the subjects with history of cancer or cardiovascular diseases (stroke, myocardial infarction and angina pectoris) (*n* = 1580). This study was approved by the Ethics Review Committee of Yamagata University Faculty of Medicine (Approval No. 2019–391), and it was conducted in accordance with the Declaration of Helsinki.

### Information on yogurt and milk intake and baseline characteristics

We collected information on yogurt and milk intake at the baseline survey using the short food frequency questionnaire (FFQ) with high reproducibility and validity reported [13]. In the questionnaire frequency of yogurt and milk intake was assessed via a single-item question: “How often do you have yogurt?” and “How often do you drink milk?”. We provided six possible answers, namely, 1 or more times/day, 5–6 times/week, 3–4 times/week, 1–2 times/week, 1–3 times/month, and < 1 time/month. Then, we categorized the frequency of yogurt and milk into four groups as none (< 1/month), low (1–3/month), moderate (1–6/week), and high (> 1/day) intake.

Data on baseline characteristics, such as age, sex, current smoking status, alcohol consumption, education period and various laboratory parameters, were collected. The education period was categorized in to three groups (< 9 years: primary or junior high school, 10–12 years: high school, and > 13 years: college or higher). We ascertained the presence of hypertension, diabetes, and dyslipidemia using the information on laboratory data and medications according to the definitions used in previous studies [12, 14].

### Outcomes

All subjects were followed up from 2009 to 2018 (9 years). Information on mortality was obtained from the certificate of residence. Information of the cause of death was collected from death certificate between 2009 and 2016 (7 years), and classified based on the International Statistical Classification of Diseases and Related Health Problems, 10th Revision (ICD-10) codes. Cardiovascular mortality was defined as death due to circulatory system disorder (ICD-10 codes I00–I99), and cancer-related mortality was defined as death due to cancer (ICD-10 codes C00–D48).

### Statistical analysis

Analysis of variance and chi-square tests were used for continuous and categorical variables, respectively, to compare the groups. Survival time analysis was performed using the Kaplan–Meier method for the association between yogurt/milk intake and mortality. To examine this association, Cox proportional hazards analysis was performed, adjusting for background factors including age, sex, smoking status, alcohol consumption, body mass index (BMI), hypertension, diabetes, and education period. The significance level for each test was set at *P* < 0.05. All statistical analyses were preformed using JMP Software 14.2 for Windows (SAS Institute Japan Ltd., Tokyo, Japan).

## Results

### Baseline characteristics of the study subjects

A total of 14,264 subjects (5323 males and 8941 females) with a mean age of 61.5 years were analyzed. The baseline characteristics of the subjects are shown in Table 1. Overall, the prevalence of smoking, alcohol consumption, hypertension, diabetes mellitus, and dyslipidemia were 12.0, 46.0, 41.2, 9.5, and 50.4%, respectively. The prevalence of none, low, moderate, and high intake of yogurt and milk was 19.9, 17.8, 38.3 and 24.0% for yogurt and 19.0, 12.3, 36.8, and 32.0% for milk, respectively. Yogurt and milk intake increased with age, but decreased with increased prevalence of males, smoking, and alcohol consumption.

**Table 1:** Characteristics of study subjects

	Total	None (< 1/month)	Low (1–3/month)	Moderate (1–6/week)	High (> 1/day)	*P*-value
		Yogurt intake				
Number (%)	14,264 (100)	2844 (19.9)	2535 (17.8)	5459 (38.3)	3426 (24.0)	
Male	37.3	64.8	38.8	26.8	30.1	< 0.01
Age, years	61.5 ± 8.7	61.8 ± 8.8	59.8 ± 9.1	61.3 ± 8.6	63.1 ± 8.0	< 0.01
Body mass index, kg/m^2^	23.0 ± 3.2	23.2 ± 3.2	23.2 ± 3.3	23.0 ± 3.2	22.8 ± 3.1	< 0.01
Smoking, %	12.0	24.5	15.5	8.0	5.5	< 0.01
Alcohol consumption, %	46.0	61.9	49.1	39.9	40.0	< 0.01
Hypertension, %	41.2	48.4	39.2	39.1	40.1	< 0.01
Diabetes mellitus, %	9.5	11.0	8.9	9.3	9.2	0.04
Dyslipidemia, %	50.4	51.5	49.5	49.9	51.1	0.34
Education period, % < 9 / 10–12 / > 13 years	12.9/70.0/17.2	15.9/68.2/15.9	12.9/71.1/16.0	11.5/71.7/16.8	12.5/67.9/19.7	< 0.01
		Milk intake				
Number (%)	14,264 (100)	2705 (19.0)	1748 (12.3)	5251 (36.8)	4560 (32.0)	
Male	37.3	45.3	43.0	34.4	33.8	< 0.01
Age, years	61.5 ± 8.7	60.7 ± 8.9	60.0 ± 9.2	61.5 ± 8.5	62.7 ± 8.3	< 0.01
Body mass index, kg/m^2^	23.0 ± 3.2	22.8 ± 3.2	23.2 ± 3.2	23.2 ± 3.2	22.9 ± 3.1	< 0.01
Smoking, %	12.0	19.3	15.2	10.4	8.3	< 0.01
Alcohol consumption, %	46.0	52.5	51.0	44.9	41.3	< 0.01
Hypertension, %	41.2	42.3	41.2	41.3	40.6	0.59
Diabetes mellitus, %	9.5	8.9	9.4	10.0	9.4	0.43
Dyslipidemia, %	50.4	48.0	48.9	50.6	52.3	< 0.01
Education period, % < 9 / 10–12 / > 13 years	12.9/70.0/17.2	13.5/69.3/17.2	13.6/69.7/16.7	12.7/70.7/16.6	12.5/69.6/17.9	0.46

**Fig. 1:**
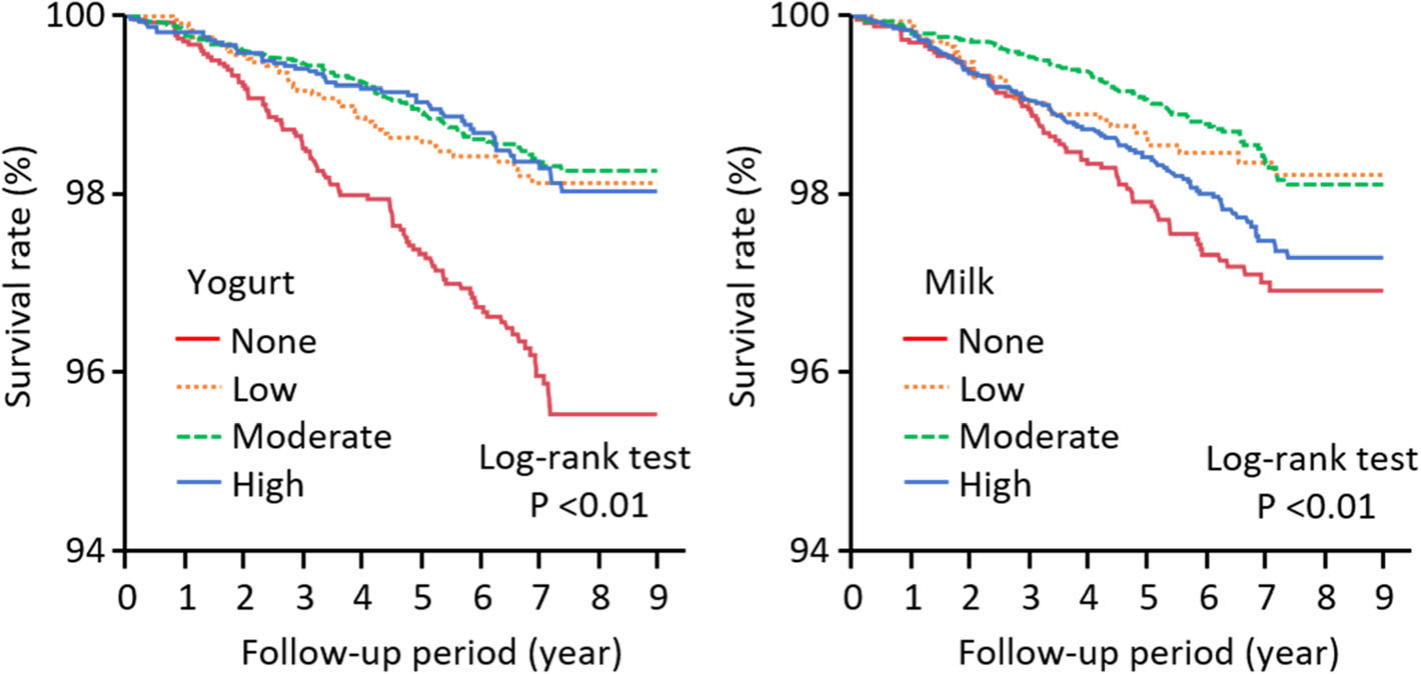
Association between the frequency of yogurt and milk intake and total mortality

During the 9-years follow-up (median 6.5 years), there were 265 total deaths. By the cause of death, there were 40 cardiovascular and 90 cancer-related deaths during the 7-year follow-up (median 4.5 years). Kaplan–Meier analysis showed that the survival probability in the high/moderate/low yogurt intake and the moderate/low milk intake groups was higher than that in the none group (log-rank, *P* < 0.01; Fig. 1).

In the Cox proportional hazards analysis, the unadjusted hazard ratios (HR) of yogurt intake for total mortality decreased in the low intake (HR: 0.45, 95% confidence interval [CI]: 0.31–0.64), the moderate intake (HR: 0.39, 95%CI: 0.29–0.52) and the high intake (HR: 0.42, 95% CI: 0.29–0.58) groups compared to the none intake group. The unadjusted HR of milk intake for total mortality significantly decreased in the low intake (HR: 0.59, 95%CI 0.37–0.92) and the moderate intake group (HR: 0.53, 95% CI: 0.38–0.74) compared to the none intake group (Table 2). In the multivariate analysis, the adjusted HR for total mortality significantly decreased in the moderate yogurt intake (HR: 0.70, 95%CI: 0.49–0.99) and the high yogurt intake (HR: 0.62, 95% CI: 0.42–0.91) and moderate milk intake (HR: 0.67, 95% CI 0.46–0.97) groups compared to the none yogurt and milk intake groups.

**Table 2:** Cox proportional hazards analysis: the association between the intake of yogurt and milk and mortality

	Person-year	Number of deaths	Incident rate (/1000 person-year)	Unadjusted		Adjusted^a^	
				HR (95%CI)	P-value	HR (95%CI)	P-value
Total mortality							
Yogurt intake							
None	17,681	99	5.6	reference		Reference	
Low	16,205	41	2.5	0.45 (0.31–0.64)	< 0.01	0.72 (0.48–1.07)	0.10
Moderate	34,767	76	2.2	0.39 (0.29–0.52)	< 0.01	0.70 (0.49–0.99)	0.04
High	21,118	49	2.3	0.42 (0.29–0.58)	< 0.01	0.62 (0.42–0.91)	0.01
Milk intake							
None	16,568	68	4.1	reference		reference	
Low	11,019	27	2.5	0.59 (0.37–0.92)	0.02	0.68 (0.41–1.09)	0.11
Moderate	33,741	74	2.2	0.53 (0.38–0.74)	< 0.01	0.67 (0.46–0.97)	0.03
High	28,443	96	3.4	0.82 (0.60–1.12)	0.22	1.06 (0.75–1.50)	0.74
Cancer-related mortality							
Yogurt intake							
None	12,166	38	3.1	reference		reference	
Low	11,214	18	1.6	0.51 (0.28–0.88)	0.02	0.90 (0.48–1.63)	0.74
Moderate	23,978	17	0.7	0.23 (0.12–0.39)	< 0.01	0.46 (0.24–0.86)	0.01
High	14,359	17	1.2	0.38 (0.21–0.66)	< 0.01	0.53 (0.27–0.99)	0.047
Milk intake							
None	11,283	27	2.4	reference		reference	
Low	7576	10	1.3	0.55 (0.25–1.09)	0.09	0.70 (0.32–1.46)	0.35
Moderate	23,360	17	0.7	0.30 (0.16–0.55)	< 0.01	0.42 (0.21–0.81)	0.01
High	19,499	36	1.8	0.77 (0.47–1.28)	0.30	1.08 (0.62–1.89)	0.80
Cardiovascular mortality							
Yogurt intake							
None	12,166	12	1.0	reference		reference	
Low	11,214	5	0.4	0.45 (0.14–1.22)	0.12	0.85 (0.26–2.49)	0.78
Moderate	23,978	15	0.6	0.64 (0.30–1.39)	0.25	1.08 (0.44–2.74)	0.87
High	14,359	8	0.6	0.56 (0.22–1.36)	0.20	1.06 (0.39–2.84)	0.91
Milk intake							
None	11,283	11	1.0	reference		reference	
Low	7576	3	0.4	0.41 (0.09–1.31)	0.14	0.52 (0.11–1.78)	0.31
Moderate	23,360	17	0.7	0.75 (0.36–1.65)	0.47	0.88 (0.38–2.18)	0.78
High	19,499	9	0.5	0.47 (0.19–1.15)	0.10	0.50 (0.18–1.35)	0.17

*CI* confidence interval, *HR* hazard ratio

^a^ adjusted for age, gender, hypertension, diabetes mellitus, smoking, alcohol consumption, body mass index, education period

Subsequently, we examined the association between yogurt and milk intake and cause-specific deaths. Similar to the total mortality, the adjusted HR for cancer-related mortality significantly decreased in the moderate yogurt intake (HR: 0.46, 95%CI: 0.24–0.86), the high yogurt intake (HR: 0.53, 95%CI: 0.27–0.99) and the moderate milk intake groups (HR: 0.42, 95% CI 0.21–0.81) compared to the none yogurt and milk intake groups (Table 2). In the overall population, the frequency of cancer-related deaths was high among subjects with lung, stomach, and pancreatic cancer. Subjects in the moderate and high yogurt intake group had a relatively lower incidence of colon, pancreatic, and lung cancer, and those in the moderate milk intake group had lower incidence of stomach and pancreatic cancer (Supplementary Table 1). However, there was no significant association between cardiovascular mortality and yogurt and milk intake in both unadjusted and adjusted Cox proportional hazards analyses (Table 2).

## Discussion

In this study we firstly showed that the moderate/high yogurt intake and the moderate milk intake are independently associated with a reduced risk of total and cancer-related mortalities in a Japanese community-based population. This finding indicates that the adequate intake of yogurt and milk might be an associated factor for good prognosis.

Neutral associations between the intake of dairy products and total, cardiovascular, and cancer-related mortalities have been reported in previous meta-analyses [1–3]. However, some studies have reported that intake of specific dairy products has protective effects—high yogurt intake was associated with a decreased risk of rectal cancer-related mortality in men [8] and high intake of dairy products was associated with a decreased risk of cardiac mortality in the Japanese [9] and Iranian population [15]. However, other reports have reported that intake of specific dairy products has harmful effects—high milk intake was associated with an increased risk of cancer-related mortality in China [16] and Sweden [17], high milk and butter intake was associated an increased risk of blood cancer-related mortality [10], and high intake of dairy products was associated with an increased risk of prostate cancer [11, 18]. The reason for the inconsistent association between intake of dairy products and prognosis has not been determined. However, differences in the classification of dairy products, the definition of high and low intake of dairy products, methods of assessing intake (frequency or amount) and the background factors of study populations may lead to inconsistencies. In this study, moderate/high yogurt intake and moderate milk intake were associated with a reduced risk of mortality, suggesting a difference in the optimal intake between yogurt and milk. These findings indicate that the amount of specific dairy products consumed might modulate the association with mortality.

The mechanism by which dairy consumption is associated with total mortality, especially cancer-related mortality, is unclear. However, the bioactive substances in dairy products such as calcium, lactoferrin, and fermentation products have been speculated to inhibit the development of various types of cancers [19]. However, insulin-like growth factor I (IGF-I) in milk has been estimated to increase the incidence of some cancers, such as prostate cancer [18]. Accordingly, the cancer-inhibiting or cancer-promoting effects may vary depending on the amount and balance of each substance in dairy products. In addition, yogurt has another possible mechanism—the involvement of live microorganisms and probiotics, such as lactic acid bacteria, which are speculated to play a protective role. It has been speculated that the anti-inflammatory effects of probiotics and their ability to inhibit the production of harmful enzymes prevent the development of colon cancer [20]. Further, it has been suggested that yogurt reduces the concentration of insulin-like growth factor I in dairy products, which promotes cancer development [21]. These experimental observations support our finding that higher yogurt intake is associated with lower cancer-related mortality.

In this study, we did not find a significant association between dairy consumption and cardiovascular mortality. However, the limited statistical power due to the small number of cardiovascular deaths may have failed to detect a significant association between them. Therefore, the insignificant association between dairy products and cardiovascular mortality is inconclusive. It needs to be confirmed by longer term observation or evaluated by other parameters such as the incidence of cardiovascular disease.

Accurate assessment of intake of yogurt and milk requires the information both of frequency and amount consumed at one time. However, it increases the burden on the study subjects and may cause another bias such as motivation and literacy. Although the food frequency questionnaire might have measurement error, the short FFQ in this study has been utilized in previous dietary intake surveys in Japan, and its high reproducibility and validity have been confirmed [13]. Therefore, this simple questionnaire could be used for a large-scale study with general population.

Our study examined the association of the intake of milk and yogurt with mortality and the cause of death in detail, with the adjustments for various background factors. Therefore, the findings of this study seem robust. However, this study has several limitations. First, owing to the observational nature of this study, it was impossible to prove a causal relationship between yogurt and milk intake and mortality. Second, yogurt and milk intake were evaluated only once at baseline and might have changed during the follow-up period. Third, there may be a selection bias because the study included subjects who underwent health screening and were relatively healthy. Fourth, we performed multivariate analyses adjusting for various established risk factors. However, there may be other confounding factors.

## Conclusions

This study showed that yogurt and milk intake was independently associated with total and cancer-related mortality among the general Japanese population. However, the association might be modulated by the amount and combination of specific dairy products consumed. Further studies are warranted to clarify the optimal intake of dairy products to improve prognosis.

## Supplementary Information

**Additional file 1: Supplementary Table 1.** Number of cancer-related deaths.

## Data Availability

The dataset of the current study is not publicly available due to ethical reasons; however, it is available from the corresponding author on reasonable request.

## Abbreviations

BMI: body mass index
CI: confidence interval
HR: hazard ratio
ICD-10: International Statistical Classification of Diseases and Related Health Problems, 10th Revision
SNPs: single nucleotide polymorphisms
