# Supplementary material for: Association between milk and yogurt intake and mortality: a community-based cohort study (Yamagata study)
Source: BMC Nutr. 2021 Jul 14;7:33. doi: 10.1186/s40795-021-00435-1 (PMC8278744; doi:10.1186/s40795-021-00435-1)
Supplement: Supplementary file 1 — Additional file 1: Supplementary Table 1. Number of cancer-related deaths. [file 40795_2021_435_MOESM1_ESM.docx]

Supplementary table 1. Number of cancer-related deaths

| ICD-10 code | Total |  | Yogurt intake | | | |  | | Milk intake | | | | |
| --- | --- | --- | --- | --- | --- | --- | --- | --- | --- | --- | --- | --- | --- |
|  |  |  | None | Low | Moderate | High | |  | | None | Low | Moderate | High |
| C07 parotid gland | 1 |  | 1 | 0 | 0 | 0 | |  | | 0 | 0 | 1 | 0 |
| C15 esophagus | 7 |  | 2 | 2 | 0 | 3 | |  | | 4 | 0 | 0 | 3 |
| C16 stomach | 11 |  | 5 | 3 | 2 | 1 | |  | | 2 | 5 | 0 | 4 |
| C18 colon | 3 |  | 2 | 1 | 0 | 0 | |  | | 1 | 0 | 1 | 1 |
| C20 rectum | 1 |  | 0 | 0 | 1 | 0 | |  | | 1 | 0 | 0 | 0 |
| C22 liver | 1 |  | 1 | 0 | 0 | 0 | |  | | 1 | 0 | 0 | 0 |
| C23 gall bladder | 4 |  | 1 | 1 | 2 | 0 | |  | | 1 | 0 | 3 | 0 |
| C24 biliary tract | 3 |  | 1 | 1 | 0 | 1 | |  | | 2 | 1 | 0 | 0 |
| C25 pancreas | 16 |  | 8 | 2 | 2 | 4 | |  | | 8 | 0 | 3 | 5 |
| C34 lung | 19 |  | 8 | 3 | 3 | 5 | |  | | 3 | 1 | 6 | 9 |
| C50 breast | 1 |  | 0 | 0 | 1 | 0 | |  | | 0 | 0 | 0 | 1 |
| C54, C55 uterus | 2 |  | 1 | 0 | 1 | 0 | |  | | 0 | 1 | 0 | 1 |
| C61 prostate | 4 |  | 1 | 1 | 1 | 1 | |  | | 0 | 1 | 1 | 2 |
| C64 kidney | 1 |  | 0 | 0 | 1 | 0 | |  | | 0 | 0 | 1 | 0 |
| C65 renal pelvis | 1 |  | 0 | 1 | 0 | 0 | |  | | 0 | 0 | 0 | 1 |
| C67 bladder | 2 |  | 1 | 0 | 0 | 1 | |  | | 0 | 1 | 0 | 1 |
| C73thyroid gland | 1 |  | 0 | 0 | 0 | 1 | |  | | 0 | 0 | 0 | 1 |
| C74 adrenal grand | 1 |  | 0 | 1 | 0 | 0 | |  | | 0 | 0 | 0 | 1 |
| C80 unknown site | 2 |  | 2 | 0 | 0 | 0 | |  | | 2 | 0 | 0 | 0 |
| C85 non-Hodgkin’s lymphoma | 2 |  | 2 | 0 | 0 | 0 | |  | | 1 | 0 | 0 | 1 |
| C90 multiple myeloma | 1 |  | 0 | 0 | 0 | 1 | |  | | 0 | 0 | 0 | 1 |
| C92 myeloid leukemia | 4 |  | 0 | 1 | 3 | 0 | |  | | 0 | 0 | 1 | 3 |
| D46 myelodysplastic syndrome | 2 |  | 1 | 1 | 0 | 0 | |  | | 1 | 0 | 0 | 1 |
